# Clinical and Operational Outcomes Associated with the Adoption of a Digital Wound Care Solution in Home Health Settings

**DOI:** 10.3390/healthcare14101387

**Published:** 2026-05-19

**Authors:** Heba Tallah Mohammed, Robert D. J. Fraser, Tameka McCabe, Amy Cassata

**Affiliations:** 1 Swift Medical Inc., Toronto, ON M5H 1A1, Canada; 2 Arthur Labatt Family School of Nursing, Western University, London, ON N6A 3K7, Canada; 3 VNS Health, New York, NY 10017, USA

**Keywords:** home health, artificial intelligence (AI), wound care, digital wound care technology, wound healing, hospitalization, value-based care, skilled nursing

## Abstract

Introduction: Wounds increase the risk of hospitalization in home health (HH) settings by up to 52%. They also consume a notable portion of HH budgets due to frequent nursing visits for wound assessment and care. To address these challenges, a U.S.-based HH enterprise adopted a Digital Wound Care Solution (DWCS) to enhance wound management and operational efficiency. This study examines the impact of integrating the DWCS into practice, focusing on clinical and operational indicators and potential cost savings. Methods: This study employed a quasi-experimental pre–post design to evaluate the impact of the DWCS on clinical and operational outcomes. Data were extracted from the DWCS and EMR databases, encompassing pre-adoption (2022) and post-adoption (2023) periods. The analysis included wound data from 16,276 patients in 2023 and 19,252 patients in 2022, covering an 8-month period (March–October) across 11 branches. The key performance indicators included skilled nursing (SN) visits per episode (VPE), time to complete SN visits, hospitalization rates, and staff optimization. Results: The adoption of the DWCS was associated with clinical and operational improvements. SN VPE decreased by 7.5%, resulting in an estimated annual savings of $1.3 million. A directional change in wound-related hospitalization rates was observed at 30 days (20.7% to 20.3%) and 60 days (32.4% to 31.5%); however, these changes did not reach statistical significance and should be interpreted as trends. The projected prevention of 200 hospitalizations with estimated annual cost savings of $3.4 million to the health system represents modeled projections based on observed directional trends rather than realized savings. A 1.9% shift in staff roles increased the utilization of licensed practical nurses with no adverse indicators identified within the scope of this analysis, saving $112,748 annually. Conclusions: The adoption of the DWCS was associated with fewer and shorter SN visits and a shift toward more LPN utilization, with anticipated reductions in costs Wound-related admissions showed downward trends but did not reach statistically significant levels. This pre–post design precludes causal attribution, and findings should be interpreted as associations rather than definitive effects of the intervention. These findings support further investigation of wound care models integrating AI within a value-based home health setting.

## 1. Introduction

Chronic wounds pose a significant health challenge in the United States, affecting approximately 6.5 million individuals annually [1]. Chronic wounds are particularly prevalent among older adults and those with underlying health conditions, such as diabetes and vascular diseases [2]. Delayed healing or poor management of these complex wounds can lead to severe complications, including infections, amputations, and increased risk of hospitalization and mortality [3,4]. For instance, between 10% and 24% of Medicare patients undergoing vascular surgery experience unplanned hospital readmissions within 30 days, while approximately 17% of patients with diabetic foot ulcers (DFU) face similar outcomes [5,6], adding a substantial financial strain on the healthcare system [3,4]. Currently, the estimated annual treatment costs for chronic wounds in the US range from $28.1 billion to $96.8 billion [7].

Home health (HH) organizations play a crucial role in the management of chronic wounds in the US [3,8,9]. As the healthcare system continues to shift towards more community-based care, outside of traditional hospital settings [10,11], the significance of HH organizations in wound management is growing. These HH organizations offer skilled nursing (SN) visits, allowing patients to receive care for wounds and related health issues in the comfort of their own homes, reducing the need for frequent hospital visits or admissions [8,12]. They closely monitor and assess the patient’s wound, which is essential for timely and appropriate interventions for a better outcome. As wound care patients account for a significant portion of home care visits, appropriate management of resources is crucial for the financial viability of home health agencies.

However, HH organizations face considerable financial and operational challenges [3,9], especially with the Medicare Home Health Prospective Payment System [8,13,14]. Medicare has traditionally reimbursed HH services in 60-day episodes, requiring organizations to offer SN visits, therapies, and wound care supplies throughout this timeframe [13]. However, with the implementation of the Patient Driven Groupings Model (PDGM) in 2020, the payment system transitioned to two 30-day payment periods within a 60-day certification period [13]. This change is intended to better align reimbursement with patient needs, minimize unnecessary services, and enhance value-based care [14]. While the 30-day payment model encourages efficiency, it presents specific challenges for managing chronic wounds. Chronic wounds often necessitate ongoing and intensive monitoring to prevent complications and ensure healing. So, in reality, the high costs associated with chronic wound management, stemming from frequent SN visits, hospitalizations due to complications, and the risk of morbidity, impose a substantial financial burden on HH organizations and the broader healthcare system. Therefore, restricting reimbursement to 30-day periods can put further strain on resources, particularly for patients with severe wounds, as agencies must carefully balance costs, SN visits, and wound care supplies within each period [15]. This financial strain may result in compromising care delivery, which can negatively impact healing outcomes and increase the risk of hospitalizations.

Further, in HH settings, registered nurses (RNs) carry much of the workload in HH wound care, often managing large caseloads under significant time constraints [16]. They are responsible for delivering comprehensive wound care, which includes tasks such as wound assessments, dressing changes, and infection monitoring, among multiple patients with varying needs [16]. This contributes to burnout and inconsistent care delivery, especially as the complexity of wound care increases [16].

More utilization of licensed practical nurses (LPNs) could be instrumental in supporting RNs [17]. Yet, LPNs frequently operate with limited access to specialized equipment, education, and resources, limiting their capacity to care for complex chronic wounds effectively [18,19]. Addressing these challenges would allow HH organizations to make better use of limited resources and improve patient outcomes and enhance the overall quality of wound care.

The emerging artificial intelligence (AI)-powered wound care technologies within clinical settings have the potential to address several of the key challenges faced by HH organizations in managing chronic wounds [18]. Research has shown that the use of AI-based wound assessment and monitoring systems can improve the accuracy of wound evaluation leading to more timely and appropriate interventions [20,21,22]. For example, Alonso et al., 2023 found that a Swift Skin and Wound^®^, an AI-powered digital wound care solution (DWCS), was able to assess wound size, with a high degree of accuracy surpassing traditional clinical assessment [21]. This capability can help overcome the limitations faced by nurses who may have limited training or resources for comprehensive wound assessment.

Moreover, AI improves communication within interdisciplinary teams by integrating data into centralized platforms, such as dashboards. This enables seamless collaboration among physicians, nurses, and therapists, ultimately reducing delays in care, and promoting consistent management strategies [19]. Additionally, AI supports value-based payment models by ensuring efficient and effective use of resources. A pilot survey study at a skilled nursing facility reported that 89% of surveyed clinicians believed the DWCS fostered better interdisciplinary communication and made it easier to collaborate on complicated wounds and adjustment of wound management plans [23,24].

By providing timely and accurate assessments along with enhanced communication, these technologies can potentially help reduce the reliance on frequent SN visits while still maintaining high-quality care [19].

The DWCS utilized in this research (Swift Skin and Wound^®^) is an AI-powered mobile and web-based platform designed to standardize wound evaluation and documentation. At the point of care, clinicians capture calibrated wound images, and the DWCS then performs automated segmentation, wound measurements, and tissue characterization. The wound image and documentation are uploaded automatically to an integrated EMR. Alternatively, records can be exported as a PDF file for a manual upload into the EMR, where direct integration is not available. A branch-level dashboard aggregates longitudinal wound data. Current standard practice is to manually measure with a paper ruler and capture notes without images or centralized reporting visibility. As such, the DWCS differed from traditional practice in signifying the difference between having automated and reproducible measurements, image-anchored documentation, and real-time oversight, all of which may have factored into the observed results.

By bridging gaps related to workforce capacity, payer constraints, and care coordination, AI has the potential to revolutionize wound care delivery in HH settings while maintaining care quality [20,25].

Despite growing interest in AI-enabled wound care, real-world evidence on the clinical and operational impact of DWCS adoption in home health settings remains limited. This quasi-experimental, pre–post comparative study addresses this gap by investigating the impact of incorporating the DWCS into a comprehensive wound care program within an enterprise home health organization. The assessment focused on both clinical and operational indicators, alongside the associated cost savings associated with the adoption of DWCS in a large U.S. home health enterprise, with a particular focus on outcomes that have received limited attention in the existing literature, including skilled nursing visit efficiency, workforce optimization, and wound-related hospitalization trends. Clinical outcomes measured the hospitalization rates due to a wound at 30 and 60 days. On the operational side, the study evaluated the average number of SN visits per 60-day wound episode, the average duration of each nursing visit, and the effective utilization of staff resources.

## 2. Methods

### 2.1. Study Design and Data Sources

This study employed a one-group pretest-posttest quasi-experimental (pre–post comparative) design to assess the impact of integrating the Digital Wound Care System (DWCS) into clinical practice at a large HH organization in the US. As an inherent limitation of this design, the absence of a concurrent control group means that the influence of temporal trends or external contextual factors on the observed outcomes cannot be ruled out, and causal inference is therefore limited.

The analysis focused on evaluating clinical and operational performance indicators as well as associated cost savings before and after the adoption of the DWCS. Data collection spanned across two time periods March-Oct: pre-adoption (2022) and post-adoption (2023) across 11 branches of the HH enterprise.

The study utilized data from Home Care Home Base (HCHB) Electronic Medical Record (EMR). HCHB platform offers a comprehensive overview of patient demographics and wound characteristics. This includes key details such as patients’ age, sex, the start date of care, 60-day episode start and end dates, as well as the types and stages of wounds. Furthermore, the system records the date and duration of each evaluation within the 60-day wound episode. It also captures information about the clinicians’ disciplines and service codes, hospitalization details—including dates and reasons for hospitalization—and various payer types. For data extraction, the HH data team filtered the records within the study periods to focus specifically on patients with wounds within the HCHB Integumentary Command Center, a centralized hub for skin assessment and care data.

### 2.2. Study Population and Setting

The study and analysis focused on wound assessments that met specific inclusion criteria. To be included, records of patients aged 18 or older were required to show wound-related diagnoses (both primary and secondary) documented in the EMR during the designated 8-month period (March to October) for both years from the participating branches. For the post-adoption period of March to October 2023, wounds had to be assessed and managed using the DWCS at the 11 participating branches. DWCS use was defined as documented utilization of the application for wound assessment and documentation during the episode.

Patients were not included in the analysis if patients’ wound data was incomplete or if their care episodes fell outside the specified study period. Additionally, patients at the adoption branches who did not utilize the DWCS for wound care during the study period were excluded in the post-adoption analysis. This exclusion applied to those with closed surgical wounds, external fixators, bruises, cellulitis, and extensive diffuse dermatological conditions.

The study gathered and analyzed data from a total of 19,252 patients in 2022 (pre-adoption) and 16,276 patients in 2023 (post-adoption), all of whom received care at the 11 home health branches that implemented the DWCS.

The reduction in patient volume between periods is partly attributable to the post-adoption eligibility criteria described above, as well as broader post-pandemic shifts in home health referral patterns and utilization trends that may have occurred independently of the intervention. Importantly, wound type distribution and patient demographics were comparable across both periods with no statistically significant differences (*p* > 0.05, Results Table 1), supporting the interpretation that this cohort size difference did not materially influence the observed outcomes.

### 2.3. Data Collection and Management

The study analyzed changes in clinical and operational outcomes by comparing data from both pre- and post-adoption periods across 11 adoption sites. In March 2023, the HH organization’s research and data team independently extracted all necessary wound patient information from the HCHB EMR based on wound start dates and medical record numbers (MRNs) for the participating branches. To ensure the dataset’s completeness and accuracy, quality assurance procedures were implemented. Following consistent filters and procedures, the eligible wound data was extracted for both pre- and post-adoption periods and subsequently de-identified for analysis by the evaluation team from the DWCS. Each patient was assigned a unique study and episode ID number, and no MRN-study ID was shared to maintain patient confidentiality.

The de-identified dataset included essential wound assessment variables, such as patient characteristics, episode ID, referral date, branch code, wound type, classification, anatomical location, wound care start date, effective care date, episode start and end dates, wound status, primary diagnosis, visit start and end times, visit duration, service code and description, discipline code, payor type, hospitalization date, and reasons for hospitalization. This data was securely shared with the evaluation team in an Excel spreadsheet through the secure platorm-Sync.com.

This study was conducted in accordance with the Declaration of Helsinki, regarding research involving human subjects. The analysis of the anonymized wound data set does not involve any interventions or affect the care provided; however, it has the potential to enhance our understanding of wounds and inform better wound care treatments.

The study obtained an exemption for ethics review from Pearl IRB, LLC, an independent institutional review board (ID: 2023-0100).

### 2.4. Statistical Analysis

The wound assessment data were analyzed using the Statistical Package for Social Sciences (SPSS; IBM Corp, Armonk, NY, USA. Version 29; 2024). The analysis included conducting descriptive statistics for numeric and categorical variables to describe patient demographics and wound characteristics. Summary statistics were illustrated as frequencies expressed in percentages (%) or as mean values and standard deviation (SD).

Bivariate analyses were performed to explore relationships between different variables. A Student’s sample *t*-test was employed to investigate whether there was a statistically significant mean difference in the average number of SN visits per episode (VPE), and the average time to complete a SN visit when comparing the assessment years of 2023 and 2022. To ensure the statistical tests’ validity, the data’s normality was assessed using the Shapiro–Wilk test, while the Levene test was utilized to evaluate the homogeneity of variances. The analyses showed that average number of SN as well as the duration for the participating branches across the pre–post periods were normally distributed, as assessed by Shapiro–Wilk’s normality test (*p* > 0.05). Additionally, there was homogeneity of variances, as assessed by Levene’s test for equality of variances for both the pre and post adoption periods (*p* = 0.236).

The analyses have been done at the patient level, with branch serving as the unit of implementation instead of being the unit of analysis. The hierarchical structure of the patients across 11 branches brings in some possible clustering effect, which has not been considered in this bivariate analysis. Multilevel modeling would have helped overcome the issue of branch-level clustering; however, considering the limited number of branch-level clusters (*n* = 11), the statistical power required for reliable random-effects estimation would be limited, risking model instability.

Additionally, a Chi-square test was conducted to compare the proportions of hospitalization due to wound at 30 and 60 days post admission, as well as the proportion of wound assessments conducted by RNs versus LPNs.

A 95% confidence interval (CI) was reported, and the significance level was set at a *p* value of less than 0.05.

### 2.5. Key Performance Indicators (KPIs)

The analysis concentrated on key clinical and operational performance indicators (KPIs), including:

1. Skilled Nursing VPE: This metric represents the average number of SN visits required during a 60-day wound care episode. It was calculated by dividing the total number of SN visits (numerator) by the overall number of episodes managed (denominator) at the participating branches throughout the study period.
2. Time to Complete Skilled Nursing Visits: This indicator reflects the average duration, in minutes, needed to carry out SN visits during the study period. The calculation is based on the time interval from the commencement to the conclusion of each in-home visit for wounds cared for within the 60-day episode.
3. Hospitalization Rates: This indicates the proportion of patients who required hospital admissions due to complications related to wound care, assessed at both 30 and 60 days post-admission.
4. Staff Optimization: This involves the number of evaluations conducted by RNs and LPNs to evaluate shifts in provider’s roles across the years.

An Economic Impact Analysis was performed to evaluate the financial impact of adopting the DWCS. This analysis included quantifying savings related to a decrease in the number and duration of SN visits, improved staff efficiency, and reduced hospitalization rates. Acquisition/maintenance costs of the DWCS and patient-level outcomes were not included.

All cost estimates derived from this analysis represent modeled projections based on observed utilization changes and published unit cost benchmarks. They should be interpreted as an economic impact framework rather than a complete cost–benefit analysis, and realized savings may differ based on implementation, operational, and payer-specific factors.

## 3. Results

### 3.1. Overall Characteristics

From March to October in 2022 and 2023, participating branches provided care for 25,570 and 22,193 wound care episodes, respectively, admitting 19,252 patients in 2022 and 16,276 in 2023. Surgical wounds were the most common in both years, comprising 48.4% of cases in 2022 and 46.8% in 2023, followed by pressure injuries (18.9% in 2022, 20.5% in 2023) and venous wounds (6.8% in 2022, 9.2% in 2023). Across both years, most patients were female (55.9% in 2022, 57.3% in 2023), with an average age of approximately 74 years (Table 1).

**Table 1:** Overall characteristics of wound records at adopted branches in 2022 and 2023.

	Adopted Branches March–October 2022	Adopted Branches March–October 2023
Number of episodes managed at participating branches	25,570	22,193
Number of patients admitted for wound care	19,252	16,276
Wound types Arterial Ulcer Burn Diabetic Ulcer Pressure Injury Skin Tear Surgical Wound Trauma Venous Other *	180 (0.5%) 172 (0.4%) 1280 (3.3%) 7295 (18.9%) 793 (2.1%) 18,704 (48.4%) 1489 (3.9%) 2633 (6.8%) 6061 (15.7%)	264 (0.7%) 59 (0.4%) 1583 (4.2%) 7803 (20.5%) 1096 (2.9%) 17,811 (46.8%) 1192 (3.1%) 3508 (9.2%) 4668 (12.2%)
Sex Male Female	8491 (44.1%) 10,761 (55.9%)	6950 (42.7%) 9326 (57.3%)
Age (Mean ± SD)	74.9 ± 15.9	73.3 ± 14.7

* Other Types of wounds: Abrasion, laceration, blisters, seroma, carcinoma, hematoma.

Overall, there were no statistically significant differences (*p* > 0.05) between the distribution of wounds assessed in 2022 and 2023, indicating a fairly comparable distribution of wound types and patient characteristics across both years.

The mechanisms through which the DWCS may have contributed to the observed operational changes, including automated wound measurement, image-anchored documentation, and dashboard-enabled oversight, are covered in Section 4.

### 3.2. Reduction in Average Number of Skilled Nursing Home Care Visits per 60-Day Episode

**Figure 1:**
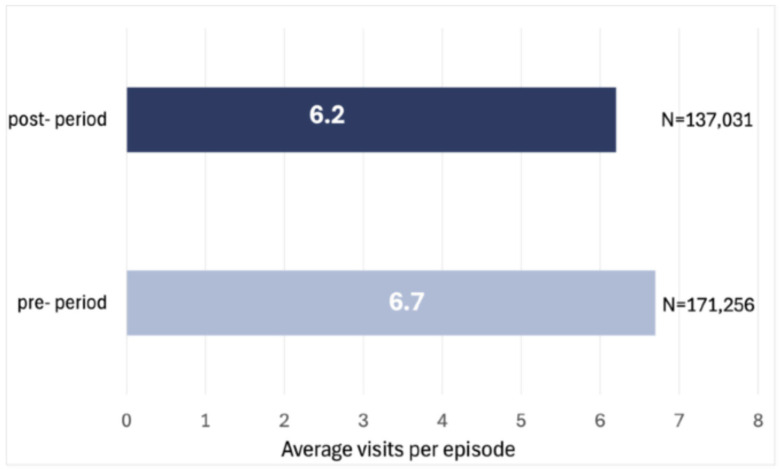
Average number of skilled nursing in-home visits per episode at adopted branches in 2022 and 2023.

The adoption of the DWCS across branches resulted in a 7.5% decline in the total number of SN VPE compared to the pre-adoption period (from an average of 6.7 VPE in 2022 to 6.2 in 2023; *p* < 0.001) (Figure 1). This reduction suggests a considerable shift in service delivery. Although the reduction of 0.5 visits per episode might seem modest, the importance of such value is apparent in a large context. So, while the absolute difference per episode is modest, its cumulative effect across the full volume of episodes is operationally meaningful and should be interpreted within that context. In total, for all 22,193 episodes during the post-adoption phase, a reduction in visit frequency by 0.5 per episode amounts to about 11,540 fewer visits, which makes a significant difference in resource usage and cost savings with no adverse care indicators identified within the scope of this analysis, though direct quality-of-care outcomes were not measured.

Additionally, in 2022, LPNs completed 19,351 visits, accounting for 11.3% of all SN visits. In contrast, RNs conducted 151,905 visits, making up 88.7% of the total. LPNs averaged 0.7 VPE, while RNs had a significantly higher average of 6.0 VPE.

By 2023, the share of visits by LPNs increased slightly to 13.2% of total visits. Meanwhile, RN visits reduced their share to 86.8%. This shift led to an increase in the weighted average VPE for LPNs to 0.8 and a decrease for RNs to 5.4 (Table 2). These changes reflect a notable shift in the distribution of skilled nursing visits conducted by LPNs and RNs over the two-year period. While this study did not capture quality-of-care indicators such as wound healing rates or adverse event rates, no negative care signals were identified within the scope of this analysis.

### 3.3. Reduction in Average Time to Conduct a Skilled Nursing Home Care Visit

**Figure 2:**
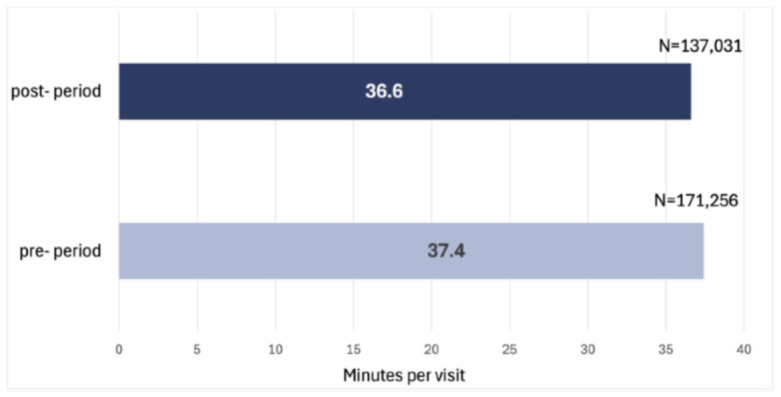
Average time to conduct a skilled nursing in-home visit at adopted branches in 2022 and 2023.

Post-adoption, the average duration of each visit decreased by 2.1%, from 37.4 min to 36.6 min (Figure 2), showing significant improvement in visit efficiency (*p* < 0.001). Even though 0.8 min reduction in visit times does seem modest, the effect is considerable when considering the full volume of visits. This means that during the eight-month adoption period, the time-saving effect of the change amounts to 114 workdays per year for the branches’ staff members.

Focusing on role-specific changes, RNs average visit duration experienced a significant decline of 4.8% in their average visit completion time, decreasing from 33.5 min in 2022 to 31.9 min in 2023 (*p* < 0.001).

### 3.4. Optimizing Staff Roles: Shifts in Provider Allocation

**Figure 3:**
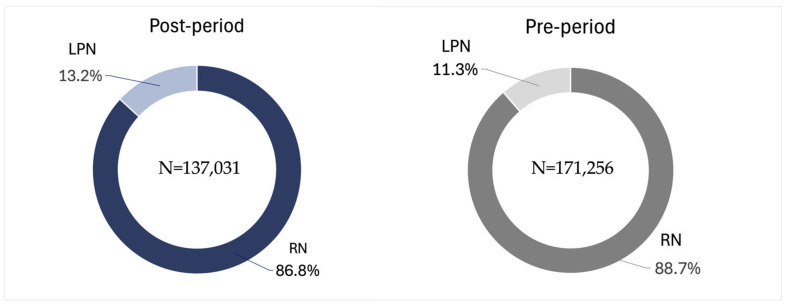
Distribution of skilled nursing home care visits between RNs and LPNs at adopted branches in 2022 and 2023.

The implementation of the DWCS has notably affected the distribution of SN home care visits between LPNs and RNs. From March to October, the total number of SN visits decreased from 171,256 in 2022 to 137,031 in 2023. During this period, LPNs increased their share of visits, rising from 11.3% (19,351 visits) in 2022 to 13.2% (18,028 visits) in 2023, a change of 1.9%. In contrast, RNs experienced a slight decline in their percentage of visits, dropping from 88.7% (151,908 visits) to 86.8% (119,003 visits), also reflecting a 1.9% decrease (Figure 3).

### 3.5. Reduction in Hospitalization Rate Due to Wound- 30 and 60-Days After Admission

**Figure 4:**
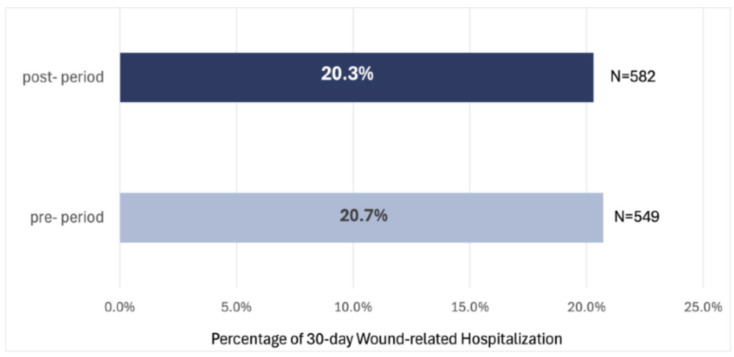
Wound-related 30-day hospitalization rates at adopted branches in 2022 and 2023.

**Figure 5:**
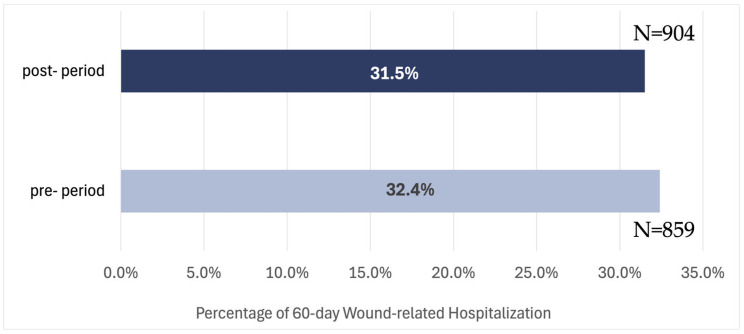
Wound-related 60-day hospitalization rates at adopted branches in 2022 and 2023.

In 2022, there were 2646 patient hospitalizations, accounting for 13.7% of total cases, while in 2023, this number was 2870, representing 17.6%. The increase in absolute hospitalization counts likely reflects changes in the patient cohort size and case-mix between the two periods rather than a deterioration in outcomes. Proportional rates are therefore the more appropriate metric for comparison, and these showed modest directional changes in the rates of wound-related hospitalizations at both 30 and 60 days. These changes were not statistically significant (*p* = 0.783 and *p* = 0.492, respectively) and should be interpreted as descriptive trends only, not as evidence of the causal effect of the DWCS. Specifically, the proportion of 30-day wound-related hospitalizations shifted from 20.7% (549 cases) to 20.3% (582 cases), a 1.9% difference (Figure 4). Similarly, 60-day wound-related hospitalizations shifted from 32.4% (859 cases) to 31.5% (904 cases), a 2.8% difference (Figure 5).

These findings highlight a downward trend in unplanned hospital admissions due to wound complications across the adopted branches.

While these directional trends are consistent with improved wound management, the absence of statistical significance and a concurrent control group precludes definitive conclusions regarding the DWCS’s contribution to these changes.

## 4. Discussion

This study examined the potential clinical, operational, and financial implications associated with the implementation of a DWCS in a HH setting. By adopting the AI-driven technology, the adoption of the DWCS was associated with a 7.5% reduction in the number of SN visits per 60-day episode, a 2.1% decrease in the average duration of each visit, a strategic 1.9% shift in visit assignments from RNs to LPNs. Directional decreases of 1.9% and 2.8% were observed in 30- and 60-day wound-related hospitalizations rates, respectively; however, these did not reach statistical significance and should not be interpreted as evidence of causal effect. Further, while the observed improvements are promising, the absence of a control group limits causal inference, and the findings should be interpreted as associations rather than definitive effects of the intervention.

The findings of this study align closely with existing research that highlights the effectiveness and advantages of implementing AI-driven solutions in healthcare, particularly in wound management [19,20,23,25]. Numerous studies published to date indicate that digital wound care technologies enhance care delivery by improving accuracy, efficiency, clinical outcomes, and fostering collaboration among care teams [19,20,23,24,25].

Notably, the observed 7.5% reduction in SN visits per 60-day episode in our study underscores the capacity of the AI systems to streamline workflow efficiency. This reduction not only lowers visit frequency but also optimizes the resources allocated to wound care, a trend supported by previous research [25]. For example, numerous studies indicate that AI-powered digital wound care technology employs advanced algorithms to streamline labor-intensive tasks such as wound documentation and measurement [26]. This technology accurately captures and standardizes wound measurements, including surface area and depth [20,21], while effectively tracking wound progression.

By enhancing precision in wound assessment and automating previously cumbersome processes for clinicians, these systems significantly help reduce the duration of assessment visits, thereby alleviating administrative burdens. This conclusion is supported by the findings of Mohammed and colleagues (2022), which showed that the AI-powered Swift Skin and Wound system provided accurate and timely automated assessments and documentation of wound dimensions and characteristics. As a result, there was a notable reduction in the time clinicians spent on wound care visits, with the study reporting an average saving of up to 50 days of clinicians’ time annually [20]. These results are in line with our observation of a 2.1% decrease in the average time spent on SN visits, equating to up to 114 days of staff time saved each year.

In addition to the capability of enhancing accuracy and speed of wound assessments, AI-driven technologies permit clinicians to track and monitor the progress of wounds remotely on dashboard platforms, enhancing the interdisciplinary collaboration of healthcare professionals on one centralized system [18,25]. Such an approach allows for the identification of patients who need immediate in-person intervention, and those patients with stable healing trajectories to receive care but with fewer visits. Capabilities like these can help reduce unnecessary SN visits per wound care episode. This advantage is realized in the current study, with the 7.5% reduction in SN visits.

Our results also align with the value-based care principles of improving efficiency, enhancing outcomes, and reducing the financial burden on healthcare organizations. All cost estimates represent modeled projections based on observed utilization changes and unit cost assumptions. Based on the observed decline in average visits per episode in 2023 vs. 2022, the 11 branches would have provided approximately 11,540 more visits by skilled nursing in the 8-month study period, had pre-adoption patterns continued. With 22,193 wound episodes occurring in 2023, and an average unit cost of $139.76 per visit, this corresponds to an estimated reduction of approximately $1.61 million in service costs during the 8-month period. Annualized, the projected difference in service costs amounts to approximately $2.42 million. The estimates indicate the modeled impact of changes in visit utilization; however, the realized cost savings may differ based on other implementation and administrative issues during the model.

Additionally, streamlining wound care workflow [20] and integrating data from various clinicians into a single platform can optimize resource allocation across the staff [27,28,29]. Providing the clinical team with effective communication and real-time guidance and insights can empower LPNs to manage less complex wound cases, thereby allowing RNs to focus on high-priority or complex cases with no adverse care indicators identified within the scope of this analysis [30].

This strategic redistribution of roles can alleviate clinician workloads and reduce costs. This has been demonstrated in our study. With the capability of AI technology in place, the HH organization reported a shift in provider allocation, with LPNs increasing their share of visits by 1.9%, suggesting effective workforce optimization. Without this favorable shift in visit allocation between RNs and LPNs, an additional 3905 visits would have been conducted by RNs. At an average cost of $78 per RN visit [31], this would have resulted in an extra annual expense of $304,619. In contrast, LPNs handled these visits at an average cost of $48 each [32], effectively mitigating some of the increased costs associated with RNs. As a result, the net annual savings from this shift is $112,748, highlighting the cost-effectiveness of optimizing nursing roles in care delivery.

Quality wound care management is essential in healthcare, as inadequate care can lead to serious complications, such as infections, amputation, and an increased risk of preventable hospital readmissions due to wound-related issues [3,4]. For healthcare providers and policymakers, reducing hospital readmission rates is one of the top priorities [33,34]. This is because preventing unnecessary readmissions improves patient outcomes and significantly lowers healthcare costs. The adoption of AI-driven digital wound care solutions has the potential of addressing this challenge [35,36,37]. By utilizing advanced analytics and algorithms, these technologies can streamline workflow, and optimize staff allocation, allowing less-trained clinicians [28] to manage simpler cases while directing the more high-risk, complex cases to the more experienced ones. This strategic focus on high-risk patients can facilitate better management strategies and early intervention, ultimately reducing complications and lowering hospital readmission rates [38,39].

Our findings are consistent with the notion that implementing DWCS may contribute to enhanced efficiency in wound care management. We observed non-significant directional decline of 1.9% in 30-day wound-related hospitalizations and 2.8% in 60-day hospitalization rates. These trends, if sustained, are estimated to correspond to 89 fewer hospitalizations within the first 30 days and 200 over the subsequent 60 days. Based on a published estimate of $13,131 per inpatient hospitalization [40], the observed directional reductions in wound-related hospitalization rates correspond to modeled projections of approximately $1,748,497 in potential annual cost avoidance for 30-day hospitalizations and approximately $3,934,119 for 60-day hospitalizations. However, given that these shifts in hospitalization rates did not reach statistical significance, these figures represent illustrative projections of potential impact rather than realized savings and should be interpreted accordingly. Furthermore, the economic analysis did not incorporate DWCS acquisition or maintenance costs, and the estimates should therefore be viewed as an economic impact framework rather than a complete cost–benefit analysis; realized cost savings may differ based on implementation, administrative, and payer-specific factors. Our results align with previous studies, which have shown that prompt and accurate wound care interventions can significantly reduce hospital readmissions and lower overall healthcare costs [7,9].

The operational, clinical, and financial efficiencies gained from adopting DWCS within the HH enterprise complement the other value-based care models’ objectives of better care, including the PDGM. Key indicators of quality care in a value-based environment include reduced SN visits, improved staffing allocation, and decreased hospitalization rates. These enhancements align with the Medicare Payment Commission’s call for a stronger alignment between healthcare efficiency and patient outcomes [14].

## 5. Limitations

The findings of this study offer valuable insights into the potential implications of implementing DWCS in home health (HH) settings. However, the research employed a retrospective pre–post comparative design that relied on existing EMR data, which may be prone to inaccuracies or missing information. Moreover, this study focused on bivariate comparisons, but did not take into account patient characteristics like the severity of wounds, and number of comorbidities that could independently affect visit frequencies and admission rates. The analysis also did not include subgroup analyses of hospitalizations by reason, nor did it adjust for individual-level confounders such as comorbidity burden, or medication use, all of which may independently influence hospitalization risk. These should be addressed in future research. Multivariate analysis should also be included in future research designs to improve causality. Further, there is a possibility of clustering effect arising from the hierarchical nature of the patients across the branches. However, considering the low number of branch-level clusters (*n* = 11), the use of multilevel modeling would not have been possible within the framework of this study. Future research that includes a higher number of implementation sites must consider hierarchical analysis techniques.

As is the case with any pre–post comparative analysis, the observed disparities between the two timeframes could be attributable to variables external to the DWCS implementation. These factors encompass natural fluctuations in home health service utilization trends during the post-pandemic recovery phase, the evolution of staffing structures, and alterations in the payer composition that may have transpired independently of the intervention.

Additionally, the study was limited to only 11 branches that adopted the DWCS within a single HH enterprise located in a large metropolitan area on the east coast of the US, which restricts the generalizability of its findings. Follow up studies can further explore similarities and differences within urban settings across the US or globally, as well as variations with rural and remote patient populations. The analysis also reflects aggregate outcomes across all wound types, and wound-type-specific effects of the DWCS were not examined. Given the considerable differences in care trajectory between surgical wounds, pressure injuries, and chronic ulcerative conditions, future studies should consider wound-type stratified analyses. Similarly, quality-of-care indicators such as wound healing rates or adverse event rates were not captured; future studies should incorporate such measures to more fully evaluate the implications of staff role shifts on care quality. Additionally, the evaluation period of eight months likely captures only the initial learning phase with the DWCS, rather than its long-term effects beyond the first year. Furthermore, potential implementation barriers associated with DWCS adoption, such as technology learning curves, equipment requirements, and data privacy considerations, were not examined in this study. These factors may influence the feasibility and scalability of real-world deployment and should be addressed in future implementation research.

To improve the applicability of these results, future longitudinal research should consider incorporating a variety of healthcare settings with different operational structures and organizational practices, as well as examining the DWCS’s impact beyond its first year of implementation. Furthermore, including a control group of branches that did not adopt the DWCS could provide more robust evidence regarding the significance of the observed outcomes. Data from non-adopting branches were not available for this analysis, which precluded a concurrent control comparison.

Furthermore, while this study emphasized key performance indicators, it did not control for potential confounding factors such as variations in clinician experience, patient socio-demographic characteristics, and adherence to management plans, all of which may have influenced clinical and operational outcomes. The study also did not explore the experience of patients not assessed with the solution. This means the findings may not entirely capture the experiences of all wound care patients within the participating branches, and utmost care should be taken when generalizing these findings to those who have not used the technology. It is worth noting that the overall usage rate of the DWCS by all eligible patients in the participating branches was not captured. Future studies should include data on adoption rates to improve the understanding of representativeness.

The financial aspect of this evaluation should be viewed as an economic impact assessment rather than a complete cost–benefit analysis. While we estimated declines in visits, staff time, and hospitalization, we did not incorporate costs of DWCS acquisition or maintenance, nor did we assess patient-level benefits. Further studies which develop clinical evaluations should include both cost measures and patient-centered outcomes to fully account for the economic evaluation.

Finally, three of the four authors are employees of Swift Medical, the developer of the DWCS under investigation, which represents a conflict of interest. To address any possible source of bias from this relationship, the EMR data were independently abstracted by the data management team at the HH facility; the anonymized data were then sent to the evaluators using an independent secure online portal (Sync.com); and independent IRB approval was sought (Pearl IRB, LLC, ID: 2023-0100). Nonetheless, despite these precautions, it is recommended that the reader take this into consideration while evaluating the findings.

## 6. Conclusions

This study provides preliminary evidence that implementing a DWCS in wound care model within HH environments is associated with improvement in both clinical and operational metrics. The utilization of this technology was associated with a reduction in the number of SN visits, the optimization of staff allocation to visits, and the directional trend toward fewer wound-related hospitalizations, though this did not reach statistical significance These associations, if confirmed in future controlled studies, may translate into meaningful financial savings for the HH organization and the wider healthcare systems. These results support the critical importance of incorporating AI-driven solutions into the operational frameworks of HH organizations to effectively manage the increasing intricacies of chronic wound care and facilitate the transition toward enduring, value-based care paradigms. The long-term effects of DWCS, however, remain to be explored in terms of quality of life and coordination of care in larger diverse populations and settings.

## Figures and Tables

**Table 2:** Average number of skilled nursing in-home visits per episode at adopted branches in 2022 and 2023.

	Adopted Branches March–October 2022	Adopted Branches March–October 2023
SN Home care visits LPN visits RN visits	171,256 19,351 (11.3%) 151,905 (88.7%)	137,031 18,028 (13.2%) 119,003 (86.8%)
Average home visits per episode LPN average VPE RN average VPE	6.7 0.7 6.0	6.2 0.8 5.4

## Data Availability

These data records are owned and governed entirely by that organization, not by the study’s authors, and were made available to the research team under a data use agreement that explicitly prohibits redistribution or unrestricted public disclosure.

## Abbreviations

The following abbreviations are used in this manuscript:

DFU	Diabetic foot ulcers
HH	Home health
SN	Skilled nursing
PDGM	Patient-Driven Groupings Model
RNs	Registered nurses
LPNs	Licensed practical nurses
AI	Artificial intelligence
DWCS	Digital wound care solution
HCHB	Home Care Home Base
EMR	Electronic Medical Record
MRNs	Medical record numbers
SD	Standard deviation
VPE	Visits per episode
